# Unlocking Antioxidant Potential in Wheat By-Products Through Microbial Bioprocessing: An Integrated Extraction–QUENCHER Assessment

**DOI:** 10.3390/molecules31142543

**Published:** 2026-07-22

**Authors:** Vanna Sanna, Valentina Tolu, Piero Pasqualino Piu, Manuela Sanna, Daniela Piras, Simonetta Fois, Angela Braca, Tonina Roggio, Pasquale Catzeddu

**Affiliations:** Porto Conte Ricerche, Località Tramariglio, 07041 Alghero, Italy; tolu@portocontericerche.it (V.T.); piu@portocontericerche.it (P.P.P.); sanna@portocontericerche.it (M.S.); pirasd@portocontericerche.it (D.P.); fois@portocontericerche.it (S.F.); braca@portocontericerche.it (A.B.); roggio@portocontericerche.it (T.R.)

**Keywords:** wheat by-products, lactic acid bacteria, yeast, sourdough fermentation, phenolics, antioxidant capacity, QUENCHER, phytate degradation

## Abstract

Wheat milling by-products are nutrient-rich sidestreams whose valorization is limited by low phenolic bioaccessibility and high phytate levels. This study investigates how sourdough fermentation using a mixed starter (*Lactiplantibacillus plantarum*, *Saccharomyces cerevisiae*, and *Wickerhamomyces anomalus*) modulates the functional properties of whole wheat germ (WWG), defatted wheat germ (DWG), defatted wheat bran (DWB), and wheat middlings (WMs), tested alone or blended with 20% semolina. Fermentation performed shifts in a substrate-dependent manner: it reduced phytic acid content by up to 60% in WWG and WMs while increasing free amino nitrogen (FAN) up to 2.6 mg/g d.b. Conversely, defatted matrices (DWG and DWB) exhibited structural resistance, showing stagnation in both phytate degradation and FAN accumulation, likely due to industrial hexane-treatment limitations. Total phenolic content (TPC) and antioxidant activities (DPPH, ABTS, FRAP) assessed via the extraction-free QUENCHER method revealed that conventional solvent extraction underestimates the bioactive potential by 40–60%. Fermentation significantly enhanced radical-scavenging activity across all matrices. However, ferric-reducing power (FRAP) increased exclusively in WWG and WMs (up to 18–19 µmol Fe (II)E/g d.b.), proving strictly dependent on phytic acid degradation. A Principal Component Analysis (PCA) revealed a clear trend toward convergence in the functional profiles of heterogeneous matrices during long-term bioprocessing. Controlled sourdough fermentation represents an effective strategy to upcycle specific cereal sidestreams into functional ingredients, though industrial defatting constraints require structural optimization.

## 1. Introduction

Wheat (*Triticum* spp.) is a cornerstone of global food security and the most widely consumed cereal crop worldwide [1]. However, industrial milling recovers only 73–77% of the grain flour, leaving large volumes of bran, germ, and middlings as underutilized sidestreams. With the wheat market estimated to reach $830.8 billion by 2026, processing generates more than 150 million tons of by-products annually [2,3].

Despite their remarkable nutritional richness, these fractions are still predominantly diverted to low-value uses, such as animal feed or biofuel production, or are discarded altogether [4]. Aligning with the Circular Economy framework and the Sustainable Development Goals (SDGs), reintegrating these nutrient-dense sidestreams into the human food chain has become a promising strategy to enhance food security and reduce the environmental footprint of the agri-food sector [5,6].

The nutritional and functional properties of wheat are tightly linked to its anatomical structure, composed of the starchy endosperm, the lipid- and vitamin-rich germ, and the protective bran layers (aleurone, hyaline, and pericarp) [7]. Milling separates these components into chemically distinct fractions: refined flour is dominated by starch and gluten proteins, whereas the outer layers are rich in dietary fiber and key phytochemicals—carotenoids, alkylresorcinols, and phenolic acids—associated with antioxidant, anti-inflammatory, and prebiotic activities [8,9]. Phenolic compounds are especially concentrated in the bran and aleurone, where they occur in free, conjugated, or cell-wall–bound forms, often covalently linked to proteins and polysaccharides like β-glucans and arabinoxylans [10]. These bioactives contribute to mitigating oxidative stress and reducing the risk of chronic diseases, including colorectal cancer, cardiovascular disease, type 2 diabetes, and obesity [11,12,13].

Because of this exceptional biochemical richness, cereal by-products have been described as “chemical goldmines” [14]. Wheat germ (WG), in particular, is rich in high-quality proteins, lipids, and vitamins, although its elevated oil content (8–14%) makes it highly susceptible to oxidation, limiting its shelf life [15].

Stabilized or defatted derivatives—such as defatted wheat germ (DWG) and Defatted Wheat Bran (DWB)—offer improved oxidative stability and greater technological versatility for sustainable upcycling [16,17,18]. However, two major biological barriers limit the health-promoting potential of these materials [19]: the low bioaccessibility of phenolic compounds bound to the cereal cell-wall matrix [20,21] and the presence of anti-nutritional factors—particularly phytates—that reduce mineral bioavailability [22].

Microbial fermentation has emerged as an effective bioprocessing strategy to overcome these limitations. Lactic acid bacteria (LAB) and yeasts act as biocatalytic systems, releasing bound phenolics and enhancing phytate hydrolysis through endogenous and microbial phytase activity [23,24,25,26]. However, the specific contribution of microbial metabolism across different milling fractions remains insufficiently characterized.

A major challenge in evaluating the nutritional value of cereal grains concerns the accurate quantification of their total antioxidant potential. Traditional methodologies typically rely on multi-step solvent extractions to separate soluble (free) and insoluble (bound) phenolic fractions, often followed by chemical (alkaline or strong acid) or enzymatic hydrolysis of the residue [27]. While effective for structural characterization, these conventional procedures are time-consuming and involve harsh chemical conditions that can degrade sensitive bioactive compounds. Furthermore, solvent-based extractions frequently underestimate the total antioxidant capacity, as they fail to capture fiber-bound phenolics [28] and overlook the synergistic effects occurring between different fractions within the native food matrix.

To overcome these limitations, the extraction-free QUENCHER (Quick, Easy, New, Cheap, and High-throughput Extraction-free) approach has been developed. This method enables a direct interaction between radical probes and the solid matrix, avoiding tedious extraction steps and providing a more realistic and comprehensive assessment of the matrix’s total reducing capacity [29,30].

To date, no study has integrated QUENCHER within long-term backslopping fermentation across multiple wheat by-products.

Based on these considerations, we hypothesized that a prolonged backslopping sourdough fermentation, utilizing a robust mixed starter culture (*Lactiplantibacillus plantarum*, *Saccharomyces cerevisiae*, and *Wickerhamomyces anomalus*), could trigger significant enzymatic degradation of anti-nutritional factors (i.e., phytates) and simultaneously enhance the bioaccessibility of bound phenolics in wheat milling sidestreams. However, we also hypothesized that the effectiveness of this bioprocess would be strictly dependent on the structural matrix, potentially limited by prior industrial treatments such as solvent defatting.

Therefore, the main objective of this research was to systematically evaluate and compare the functional and biochemical dynamics of four diverse wheat by-products (whole wheat germ (WWG), defatted wheat germ (DWG), defatted wheat bran (DWB), or wheat middlings (WMs)) subjected to a 14-day fermentation. Specifically, the study aimed to: (i) quantify the kinetics of phytic acid degradation and free amino nitrogen (FAN) accumulation; (ii) assess the evolution of total phenolic content and antioxidant activities (DPPH, ABTS, FRAP) using an extraction-free (QUENCHER) approach to avoid underestimation biases; and (iii) elucidate, through multivariate statistical analysis (PCA), how the initial matrix structure dictates the final functional profile of the upcycled ingredients.

## 2. Results and Discussion

### 2.1. Wheat By-Product Characterization

The chemical composition of the investigated wheat by-products (WWG, DWG, DWB, and WMs) prior to fermentation is reported in Table 1.

Some macro- and micro-components reveal a significant biochemical diversity, with an extensive range of values observed for ash, proteins, lipids, and fiber. Such variability is expected, considering the diverse anatomical sources (bran and germ) and processing treatments (defatted and pure).

For instance, WWG is derived from common wheat; however, its protein (15.5%), lipid (4.41%), and total dietary fiber (TDF, 34.91%) levels are different from values commonly reported in the literature for pure wheat germ [14,31]. This discrepancy is likely due to the inclusion of the outer layers of the wheat kernel, which raises the TDF while simultaneously decreasing the protein and lipid content. Conversely, DWG and DWB, derived from durum wheat germ and bran, respectively, contain low amounts of lipids (2.34% and 1.15%) as a result of the defatting process. The analytical values for WMs are consistent with established literature for durum wheat derivatives [7,31].

From a functional perspective, this compositional heterogeneity is not merely descriptive but also anticipates distinct fermentation behaviors. Specifically, the high concentrations of proteins and ash—particularly prominent in DWG—are key determinants of the substrate’s buffering capacity [18,32].

Such buffering capacity is especially relevant in sourdough systems, as it allows organic acids to accumulate without causing a proportional decrease in pH, thereby sustaining microbial activity over prolonged fermentation cycles [33].

Furthermore, the diverse distribution of TDF across the samples, peaking in WMs (46.60%), is likely to affect the structural integrity of the matrices and the overall dynamics of nutrient accessibility during fermentation [17,34]. High-fiber matrices such as WMs and DWB are also expected to contain larger pools of bound phenolics, which may become progressively mobilized during fermentation.

Overall, the distinct biochemical profiles of the four substrates provide a mechanistic basis for the substrate-dependent acidification, microbial growth, and phenolic release described in the following sections.

### 2.2. Sourdough Acidification and Microbial Growth

**Figure 1:**
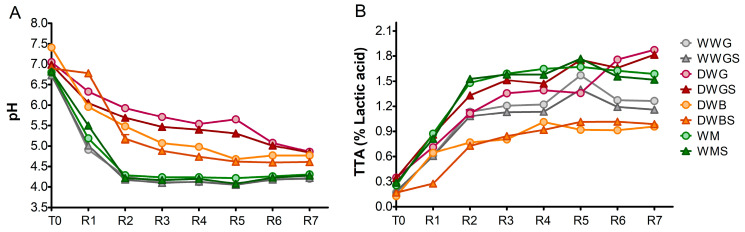
Changes of pH (**A**) and TTA values (**B**) in sourdough samples before inoculum addition (T0) and during daily refreshments (R1–R7). TTA is expressed in % lactic acid. Error bars indicate the standard deviation (SD) of three replications.

The evolution of pH and Total Titratable Acidity (TTA) in sourdough samples over the seven refreshment cycles (R1–R7) provides useful information on the biochemical transformation of the different wheat by-products, as shown in Figure 1.

Although all substrates underwent progressive acidification, the kinetics varied substantially according to their origin. The WM and WWG series (including their semolina-blended counterparts) exhibited the most rapid pH decline, reaching significantly lower values (between 4.0 and 4.2) as early as the second refreshment (Figure 1A), compared to the other matrices.

In contrast, the defatted DWB and DWG sourdoughs showed a markedly slower and statistically distinct acidification pattern, with a gradual pH decrease that stabilized only between R5 and R6.

This slower pH drop in the germ-derived matrices (DWG and DWGS), which maintained comparatively higher and statistically significant pH values during the early and mid-stages, is clarified by their TTA profiles (Figure 1B). Here, they exhibited a continuous and pronounced increase in acidity. This combination of elevated TTA and high pH indicates a strong buffering capacity, likely related to the high concentration of proteins and minerals remaining after lipid removal [32]. These components neutralize free protons, allowing organic acids to accumulate without causing a proportional decrease in pH, thereby helping to maintain microbial activity over prolonged cycles. Within this series, the incorporation of semolina promoted significantly faster initial kinetics, with DWGS exhibiting consistently higher TTA values than DWG from R1 to R5 (*p* < 0.05).

Nevertheless, the intrinsic nature of the substrate emerged as a determinant of fermentation behavior rather than the defatting treatment. This is particularly evident when comparing the two defatted matrices: the bran-derived sourdoughs (DWB) consistently exhibited the lowest TTA values (≈0.9–1.0% lactic acid at R7), whereas the germ-derived sourdoughs (DWG) supported an intense and prolonged acidification process. The marked, statistically significant increase in TTA observed in DWG after R4 (reaching ~1.4% lactic acid and peaking at approximately 1.9% lactic acid at R7) suggests that defatting not only enhances buffering capacity but may also increase the accessibility of endogenous nutrients, thereby sustaining microbial metabolism in the later stages of fermentation.

**Figure 2:**
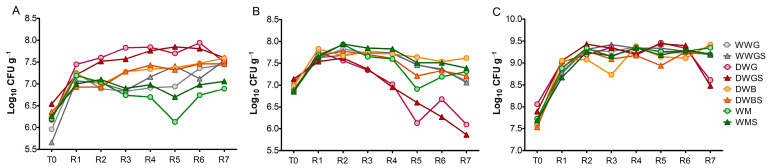
Cell density (Log_10_ CFU g^−1^) of presumptive *S. cerevisiae* (**A**), *W. anomalus* (**B**), and *L. plantarum* (**C**) in sourdough samples after daily refreshment. T0 refers to the cell density after an inoculum of the microbial starter.

To further elucidate the microbial dynamics underlying these acidification trends, LAB and yeast populations were monitored during the backslopping fermentation of wheat milling by-products, as shown in Figure 2.

Following the transition from the initial slurry to R1, *S. cerevisiae* populations stabilized between 6.9 and 8.0 Log_10_ CFU g^−1^ (Figure 2A). Defatted germ-based matrices (DWG and DWGS) supported the highest *S. cerevisiae* counts throughout the fermentation, likely due to their elevated pH (Figure 1A) and to the high concentration of proteins and minerals remaining after lipid removal [35]. Overall, the yeast population remained relatively constant, with the notable exception of WMs, which experienced a sharp drop at R5, presumably due to temporary metabolic deceleration during weekend refrigeration [36].

As shown in Figure 2B, *W. anomalus* maintained relatively high populations (around 7.5 Log_10_ CFU g^−1^) in most substrates. A progressive decline occurred in the DWG and DWGS samples after R2, dropping to minimum values at R7. Since this depletion was not observed in the whole wheat germ (WWG), it suggests that the presence of lipids in non-defatted matrices might help mitigate acid-related stress [37]. In the defatted germ, the combination of a high buffering capacity and very high TTA (approximately 1.9% lactic acid) creates a demanding environment where organic acid accumulation imposes a substantial metabolic burden, leading to the observed decline in *W. anomalus*. Conversely, *S. cerevisiae* appeared more resilient under these conditions [38], highlighting the distinct ecological niches of the two yeasts regarding organic acid tolerance.

*L. plantarum* emerged as the dominant microorganism, rapidly increasing from T0 to R1 and stabilizing between 9.0 and 9.5 Log CFU g^−1^ across most substrates (Figure 2C). This confirms that the tested wheat by-products generally provide a favorable environment for LAB, driving the overall pH reduction and TTA accumulation. Notably, however, both DWG and DWGS triggered a sharp decrease in LAB counts at R7, falling below 8.6 Log CFU g^−1^.

Beyond R1, the sourdoughs generally entered a pseudo-steady state where LAB outnumbered yeasts by approximately 2 Log units [37]. Among the substrates, defatted wheat germ proved to promote a greater growth of *S. cerevisiae* while concurrently generating acidification conditions that restricted *W. anomalus* and, in the final stage, *L. plantarum*.

### 2.3. Sourdough Metabolites

**Figure 3:**
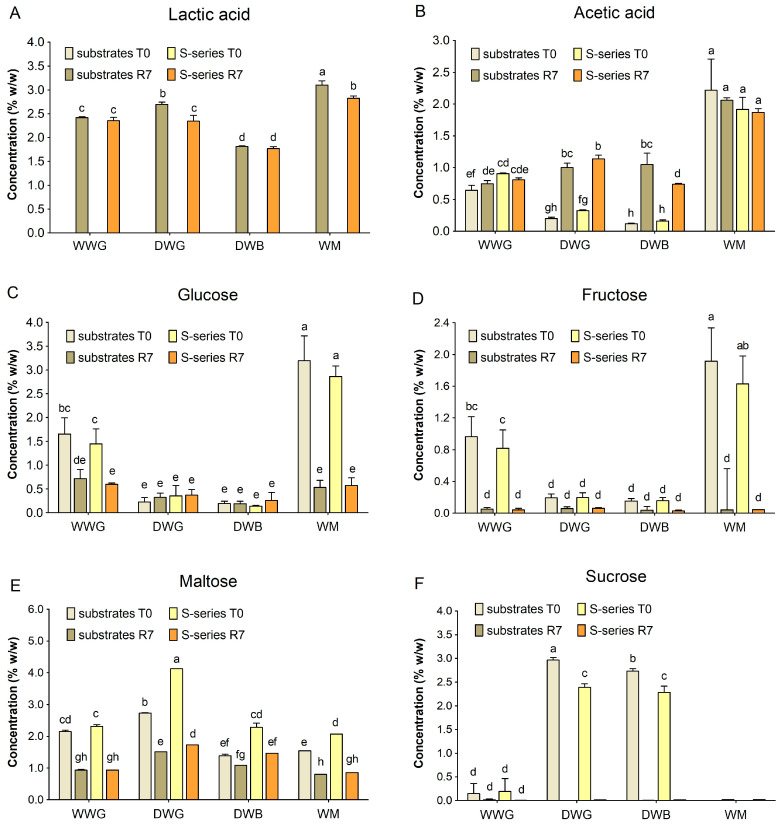
Amount of lactic acid (**A**), acetic acid (**B**), glucose (**C**), fructose (**D**), maltose (**E**), and sucrose (**F**) in WWG, DWG, DWB, and WM sourdough samples (substrates and S-series) at T0 and at R7. Bars indicate standard deviation. Different letters above the bars within the same panel indicate statistically significant differences (*p* < 0.05).

The backslopping fermentation induced marked biochemical changes across all wheat by-products. The metabolite profiles at the seventh refreshment (R7) reflect the combined influence of substrate composition, endogenous enzymatic activity, and microbial adaptation to the specific biochemical environment of each matrix (Figure 3).

A significant increase in both lactic and acetic acids was observed in most samples at R7 (Figure 3A,B), indicating active metabolism of *L. plantarum* and the associated yeasts. A baseline concentration of acetic acid was already detectable at T0 in all matrices. This initial presence is consistent with previous reports indicating that this finding may be related to grain conditioning practices aimed at reducing microbial loads on the kernel surface [39,40]. Since these by-products originate from the outermost and peripheral layers of the caryopsis, they retain residues of these conditioning treatments.

Among these substrates, WMs exhibited the most pronounced lactic acid accumulation (3.1% *w*/*w*) in line with its pH and TTA values observed (Figure 1A). This suggests that WMs provide a readily fermentable carbohydrate pool and a favorable environment for LAB proliferation, consistent with its high TDF content and intermediate protein levels.

Defatted wheat germ (DWG) displayed a distinctive lactic acid production profile, ranking second (2.7% *w*/*w*) behind WMs and reaching the highest TTA values (~1.9% lactic acid) at R7; nevertheless, it maintained a comparatively higher pH than WMs. This property is technologically relevant, as it supports prolonged microbial activity while preventing excessive acid stress.

The monitoring of fermentable sugars revealed a near-complete depletion of glucose, fructose, and maltose by R7 (Figure 3C–E). Notably, WMs exhibited exceptionally high initial concentrations of glucose (Figure 3C) and fructose (Figure 3D) at T0 compared to the other substrates, providing an abundant, readily available carbon source that correlates with its superior acidification performance. Conversely, sucrose (Figure 3F) was uniquely abundant at T0 in the DWG and DWB samples but was completely exhausted by R7, indicating rapid consumption by the sourdough microbiota.

The rapid disappearance of these sugars across all matrices suggests their utilization by LAB and yeast strains. Concurrently, the reduction in maltose (Figure 3E) reflects active amylolytic processes—either endogenous or microbially induced—that continuously supplied fermentable monosaccharides. This sustained carbohydrate turnover ensured a stable energy supply throughout the backslopping cycles, supporting the metabolic intensity observed in the acidification and microbial growth profiles.

**Figure 4:**
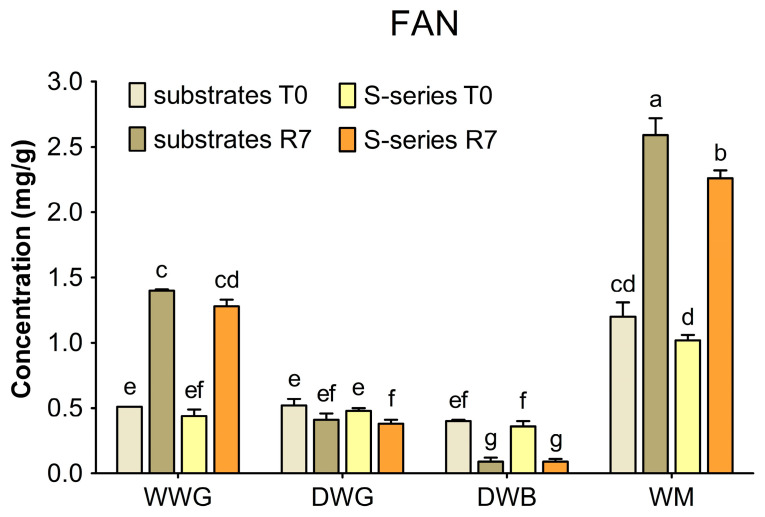
FAN concentration in WWG, DWG, DWB, and WM sourdough samples (substrates and S-series) at T0 and at R7. Bars indicate the standard deviation. Different letters above the bars indicate statistically significant differences (*p* < 0.05).

Free amino nitrogen (FAN) dynamics further highlighted substrate-specific behaviors (Figure 4).

WWG and WMs exhibited a substantial and significant increase in FAN from T0 to R7, regardless of the presence of semolina. WWG increased from approximately 0.51 mg/g d.b. to 1.40 mg/g d.b., while WMs reached the highest absolute concentration, rising from 1.20 mg/g d.b. to 2.60 mg/g d.b. This marked accumulation suggests robust proteolytic activity, likely driven by the synergy between endogenous and microbial enzymes and the inoculated microbial consortium, which efficiently hydrolyzed complex proteins into assimilable nitrogen compounds [41,42].

In contrast, the defatted matrices (DWG and DWB) showed limited or negative FAN evolution. In DWG, FAN values remained stable around 0.40–0.50 mg/g d.b. during the fermentation process, while DWB exhibited a marked decrease from 0.40 mg/g to less than 0.10 mg/g at R7. This lack of FAN accumulation is likely associated with the structural and thermal impacts of industrial hexane extraction and subsequent desolventization.

Indeed, hexane treatment is known to induce severe conformational changes and partial denaturation of both storage proteins and endogenous enzymes [43]. In agreement with established biochemical models [44], such structural modifications are expected to reduce protein solubility and accessibility to enzymatic cleavage, while simultaneous solvent- or heat-induced denaturation likely impairs the residual catalytic activity of endogenous proteases.

Moreover, the thermal stress typically applied during the desolventization step likely exacerbated this enzymatic inactivation, further limiting protein hydrolysis.

In DWB, the highly fibrous bran matrix exacerbated this limitation by physically restricting enzyme–substrate interactions. As a result, the minimal initial FAN was rapidly consumed by the starter cultures (*S. cerevisiae*, *W. anomalus*, and *L. plantarum*) to sustain their growth. In the absence of compensatory proteolytic release, this led to near-total nitrogen depletion at R7, explaining the observed slower acidification and the decline of *W. anomalus* observed in DWB and DWG, respectively.

The addition of 20% semolina (S-series) did not alter the overall trends of the respective pure substrates but resulted in significantly lower FAN values at R7 in WMS. This dilution effect reflects the lower proteolytic potential of semolina compared to nutrient-rich by-products such as germ and middlings.

Overall, the FAN trends were strongly aligned with both microbial growth and acidification kinetics. WMs and WWG, which displayed the highest FAN accumulation, also exhibited the fastest pH drop and good LAB proliferation, confirming that nitrogen availability supported sustained microbial metabolism. Conversely, the FAN decreases in DWB and DWG paralleled the slower acidification and the decline of *W. anomalus* after R2, respectively. These results suggest that nitrogen availability may have been a limiting factor in defatted matrices, where hexane extraction restricted proteolytic accessibility.

**Figure 5:**
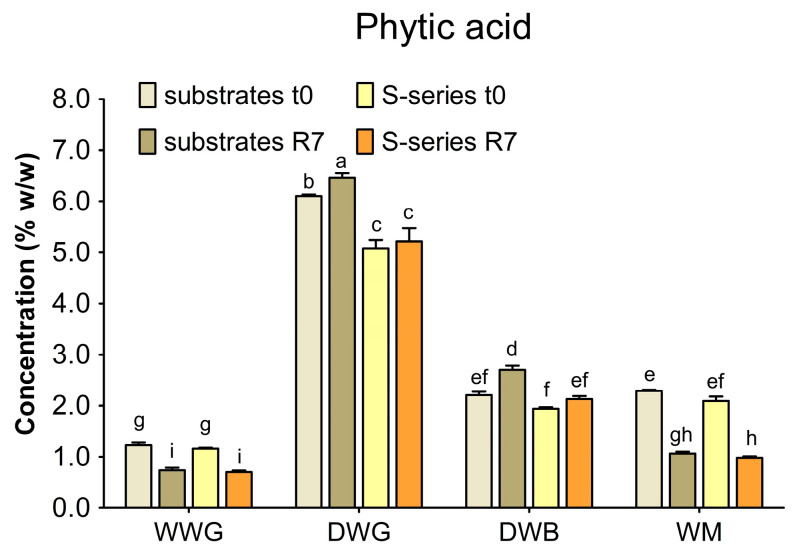
Phytic acid concentration in WWG, DWG, DWB, and WM sourdough samples (substrates and S-series) at T0 and at R7. Bars indicate the standard deviation. Different letters above the bars indicate statistically significant differences (*p* < 0.05).

At T0, phytic acid was detected in significant concentrations across all wheat by-products (Figure 5), consistent with its primary accumulation of phytates in the aleurone layer, pericarp, and germ [7].

Baseline levels varied markedly among the matrices, peaking in DWG (~6% *w*/*w*), followed by WMs (2.3% *w*/*w*), DWB (2.2% *w*/*w*), and WWG (1.2% *w*/*w*).

Despite the lower phytic acid content of the refined endosperm [10,31], the incorporation of 20% semolina (S-series) did not reduce phytate density, except in the DWG series.

The exceptionally high baseline concentration in DWG (exceeding 6% *w*/*w*), which significantly surpasses standard literature values for whole wheat germ (2.0–3.5%), can be attributed to two main factors. First, industrial hexane extraction removes the lipid fraction (~10–12% of the germ’s original mass), causing a concentration effect on the remaining non-lipid constituents on a dry weight basis. Second, as the germ is highly metabolically active, it contains a dense pool of non-phytate phosphorus (e.g., inorganic phosphate, cytoplasmic RNA, and free nucleotides). During the initial HCl extraction, these background phosphate species are co-solubilized; if they saturate the purification thresholds of the assay, they bypass the clean-up steps and artificially inflate the spectrophotometric reading [45].

After the 14-day backslopping protocol (R7), a divergent kinetic trend was observed. A sharp, statistically significant decrease in phytic acid content (up to 60%) was achieved exclusively in WWG and WMs. This depletion was driven by matrix acidification, which activated cereal endogenous phytases (acidic pH optimum between 4.5 and 5.5) working synergistically with microbial enzymes [6,23,32].

Conversely, DWG and DWB showed no apparent phytic acid reduction. This structural resistance is strongly mirrored by the FAN profiles: while WWG and WMs exhibited marked proteolysis, their defatted samples showed a complete stagnation in FAN levels. This trend is highly consistent with established structural models in the literature, which describe how the protein–polysaccharide network of defatted matrices—compacted and rigidified by industrial extraction and desolventization—can physically entrap phytates, creating severe steric hindrance that restricts access for both endogenous and microbial enzymes [46,47,48]. In DWB, this effect was further compounded by the highly fibrous nature of the bran matrix [34].

To clarify the mechanisms behind this persistence, the viability of endogenous enzymes was thoroughly investigated. To determine whether industrial defatting had permanently denatured the native phytases, an assay was conducted under optimized conditions (diluted ratio 1:10 *w*/*v*, pH 5.2, 45 °C). After 3 h, a significant decrease in phytate was observed, suggesting that endogenous phytase activity was largely retained despite the harsh industrial processing. Under sourdough fermentation conditions, these residual enzymes likely converted phytate into inorganic phosphorus; this localized accumulation might have promoted the rephosphorylation of lower inositol phosphates or exerted a feedback inhibition on the phytases, masking any apparent phytic acid reduction at R7 [49].

To identify the ultimate limiting factor during sourdough fermentation, the focus shifted to water availability. When DWG and DWB were incubated at hydration ratios mirroring real dough conditions (1:2 and 1:2.5 *w*/*v*, respectively), phytic acid levels remained unchanged compared to T0 after 3 h. Water is essential for enzyme mobility and catalytic conformation [50]. Because defatted substrates possess an extremely high protein content (Table 1), a substantial portion of the added water likely became tightly bound to the protein matrix. This compartmentalization minimized the availability of free water required for hydrolytic reactions, effectively hindering endogenous phytase activity in the fermented doughs.

Finally, the apparent phytic acid stagnation or slight increase at R7 in defatted matrices may also involve analytical artefacts and dry matter dynamics. Prolonged fermentations can release non-phytate phosphorus species that interfere with the colorimetric ammonium-molybdate reaction, inflating the phytic acid readings [45,51]. Additionally, the metabolic activity of *L. plantarum* causes a progressive loss of soluble solids and total dry matter [32]. Because the phytate network remained physically protected from degradation, the ratio of undegraded phytic acid to total dry matter shifted upward, contributing to the apparent concentration effect at R7.

Taken together, these observations suggest that industrial defatting treatments may strongly condition matrix resilience, thereby shaping both anti-nutritional degradation kinetics and the biochemical environment in which microbial metabolism occurs. Nevertheless, the present data demonstrate that long-term backslopping fermentation remains an effective bioprocessing strategy to overcome the biological barriers of specific wheat by-products, such as whole wheat germ and middlings. By reducing phytic acid content by up to 60%, this approach successfully “bio-activates” and upgrades cereal sidestreams into high-value, nutrient-dense ingredients suitable for human food upcycling [3,4].

### 2.4. Phenolic Content

**Figure 6:**
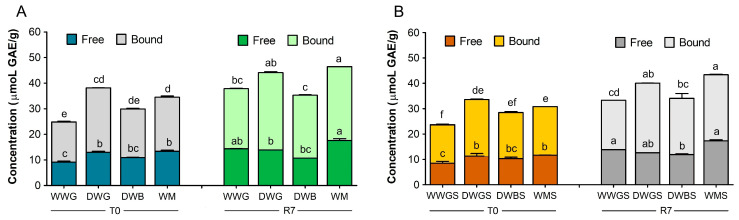
Free (darker shade) and bound (lighter shade) phenolic compound content (expressed in μmoL GAE/g d.b.) in sourdough slurries formulated without (**A**) and after mixing with 20% durum wheat semolina (**B**). For both panels, the group of bars on the left represents the systems before inoculum (T0), while the group on the right represents the sourdoughs after seven consecutive refreshments (R7). The bound phenolic fraction was determined directly on the solid matrices via the QUENCHER method. Error bars indicate the standard deviation (SD) of two independent analytical replications. Different letters above the error bars indicate statistically significant differences for total phenolic content (Q-TPC), while different letters within the darker bars indicate significant differences for the free phenolic fraction (*p* < 0.05).

Figure 6 illustrates the concentration of free and bound phenolic compounds (expressed in μmoL GAE/g d.b.) across the different wheat milling by-products before fermentation (T0) and after seven refreshments (R7), distinguishing pure matrices (Figure 6A) from their 20% semolina blends (Figure 6B).

Across nearly all substrates, long-term fermentation induced a significant increase in total phenolic content, with wheat middlings (WMs and WMS) reaching the highest value at R7 (46.4 ± 0.67 and 43.4 ± 0.41 μmoL GAE/g d.b., respectively) (Figure 6A,B). Interestingly, this enhancement was characterized by a simultaneous increase in both free and bound phenolic fractions. Rather than a simplified shift from bound to free forms, these results suggest that sourdough fermentation enhanced the overall extractability and chemical reactivity of the complex phenolic structures within the matrix, potentially driven by microbially induced structural modifications or the de novo synthesis of antioxidant compounds by the fermenting microbiota [32].

To quantify the mobilization of these compounds, the release efficiency (RE%) was calculated and summarized in Table 2. At T0, the by-products exhibited distinct phenolic profiles: WMs showed the highest initial free phenolic content (13.37 μmoL GAE/g d.b.), closely followed by DWG (12.96 μmoL GAE/g d.b.). However, DWG was characterized by the largest reservoir of bound phenolics (12.21 μmoL GAE/g d.b.). This is primarily attributable to the specific histological origin of the germ, which is naturally rich in cell-wall-bound phytochemicals, further concentrated by the lipid removal process [18]. Conversely, pure DWB (8.09 μmoL GAE/g d.b.) and WMs (7.78 μmoL GAE/g d.b.), presented significantly lower initial bound fractions, reflecting their different tissue origins and cell-wall architectures [24], suggesting that tissue composition, rather than processing alone, governs the phenolic distribution.

The transition from T0 to R7 highlights the strong capacity of the microbial consortium (*S. cerevisiae*, *W. anomalus*, and *L. plantarum*) to promote the release of matrix-bound phenolics. This deep biotransformation is likely facilitated by the synchronous action of microbial and endogenous hydrolytic enzymes—such as feruloyl esterases, cellulases, and xylanases—which partially degrade the cereal cell-wall architecture by cleaving ester linkages between phenolic acids (primarily ferulic acid) and arabinoxylan chains [52,53].

A comparative analysis between pure by-products and their respective semolina blends highlights substrate-dependent synergistic effects rather than a simple dilution effect. As visually captured by the shifting profiles between Figure 6A,B, and quantified by the RE% values in Table 2, the incorporation of 20% semolina exerted a major, unpredicted impact on specific matrices. The most remarkable synergy occurred in WMs, where the RE% increased from 54.5% (WMs) to 75.5% (WMS). This suggests that semolina provides readily fermentable carbohydrates that stimulate microbial metabolism and subsequent enzyme production, thereby optimizing phenolic release [52,53].

Crucially, a pivotal synergistic effect was observed for DWB: while pure DWB showed no net mobilization (RE = −2.7%), its blended counterpart (DWBS) achieved a positive RE of 20.3%, suggesting that the co-substrate may have redirected microbial metabolism towards cell-wall deconstruction [52,53].

Defatted wheat germ (DWG) showed a minor yet positive response to semolina blending, with the RE% increasing from 7.7% in the pure matrix to 11.8% in DWGS. This limited enhancement suggests that, while the addition of semolina slightly boosted microbial enzymatic activity, the massive and highly stable initial bound phenolic reservoir of the defatted germ (12.21 μmoL GAE/g d.b.) remained mostly resilient to further breakdown.

Conversely, semolina had a negligible impact on whole wheat germ systems (WWG, 78.9% vs. WWGS, 78.8%), confirming that the native germ matrix already provides an optimal nutrient environment for maximal enzymatic expression [1,31,34].

A major analytical strength of this work is the integration of conventional Folin–Ciocalteu extraction with the solid-state QUENCHER approach. Because bound phenolics are typically underestimated by solvent extraction [29], the QUENCHER method revealed that matrices such as WMs and DWG possess a substantially higher functional density than previously reported [54], while also aligning with green chemistry principles by reducing solvent use [55].

In conclusion, while DWG retains a high absolute phenolic content due to concentration effects from defatting [18], WWG and WM matrices demonstrated the greatest functional responsiveness to fermentation, achieving the most effective bioactivation. By enhancing the free-to-bound ratio and unlocking fiber- and mineral-associated phenolics, long-term fermentation emerges as a powerful strategy to upgrade cereal sidestreams into high-value functional ingredients [3,19].

### 2.5. Antioxidant Activity

The functional impact of phenolic mobilization was assessed through three complementary QUENCHER assays—DPPH, ABTS, and FRAP—allowing direct quantification of total antioxidant activity from the solid matrix. This direct approach captures both soluble and insoluble fractions, overcoming the extraction biases of conventional liquid assays [30]. Traditional methods, which rely on multi-step extraction and hydrolysis, are laborious, may degrade sensitive compounds, and systematically fail to recover fiber-bound phenolics—leading to underestimations of 40–60% in wheat co-products [29,54]. In contrast, QUENCHER measures the total reducing capacity at the solid–matrix interface, providing a more comprehensive and realistic assessment of antioxidant potential, particularly in cereal-based systems where most antioxidants are bound to the insoluble matrix [56].

However, a balanced evaluation of the QUENCHER methodology requires acknowledging its inherent limitations. While it effectively circumvents extraction biases, the solid-state reaction is highly susceptible to complex matrix effects, where the physical structure and compactness of the solid residue can sterically hinder the accessibility of radicals to certain antioxidant sites [57]. Furthermore, like all spectrophotometric screening tools, QUENCHER assays lack compound specificity. They provide a collective, indirect measure of the total reducing and scavenging capacity at the interface but remain fundamentally unable to distinguish individual antioxidant contributors or provide direct biochemical evidence regarding the precise molecular identity of the active species [58,59].

**Figure 7:**
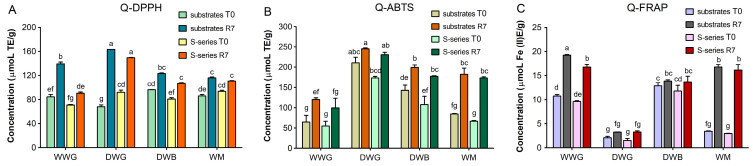
Changes in DPPH (**A**), ABTS (**B**), and FRAP (**C**) QUENCHER results of slurries containing by-products alone (substrates) and mixed with semolina (S-series), before inoculum (T0), and after seven refreshments (R7). Estimated means were expressed as μmoL TE/g d.b. for DPPH and ABTS, and as μmoL Fe(II)E/g d.b. for FRAP, respectively. Error bars indicate the SD of two replications. Different letters above the bars within the same panel indicate statistically significant differences (*p* < 0.05).

Across all substrates, fermentation markedly enhanced radical-scavenging capacity (Figure 7). In the DPPH assay (Panel A), all substrates showed increased hydrogen-donating ability at R7, with DWG exhibiting the strongest response (from ~70 to over 160 μmoL TE/g d.b.). This 2.5-fold increase reflects the extensive enzymatic deconstruction of the germ cell-wall network driven by the synergistic hydrolytic activity of *L. plantarum* and *Saccharomyces* species. Their combined cellulase, xylanase, and esterase activities progressively disrupt the cereal matrix during prolonged fermentations, thereby releasing a dense pool of antioxidant moieties and bound phenolic compounds [23,60,61].

This prominent response is strictly intertwined with the native chemical composition of DWG (Table 1); the removal of the lipid fraction increases the relative abundance of proteins and concentrates the non-polar and bound phenolic constituents within the remaining solid matrix, thereby enhancing the radical-scavenging reactivity once these compounds are released during microbial fermentation [62].

The Q-ABTS assay (Panel B) confirmed these trends, yielding higher absolute values due to its broader sensitivity to both hydrophilic and lipophilic antioxidants [63]. DWG reached approximately 250 μmoL TE/g d.b., while DWB nearly doubled its baseline activity. The enhanced reactivity toward ABTS^•+^ is consistent with the release of hydroxycinnamic acids, particularly ferulic acid, whose electron-donating properties are favored in aqueous–alcoholic environments [64].

The Q-FRAP assay (Panel C) provided additional mechanistic insight into the reducing capacity of the fermented matrices. Unlike DPPH and ABTS, FRAP values were strongly governed by phytic acid dynamics and, consequently, by the acidification profiles of each substrate.

Although DWG exhibited the highest radical-scavenging activity in both Q-DPPH and Q-ABTS, reflecting its rich native phenolic content, its ferric-reducing performance remained markedly suppressed at all stages, with values close to zero.

This discrepancy is explained by the exceptionally high phytic acid content of DWG and its structural resistance to enzymatic degradation. As demonstrated in previous sections, the phytate content in DWG remained essentially intact after 14 days of fermentation due to steric hindrance and limited phytase accessibility.

Furthermore, the structural compaction and partial protein denaturation induced by the desolventization process (hexane treatment) restricted the availability of free water within the DWG matrix, potentially hindering enzymatic unmasking [65]. Since FRAP relies on the reduction in free Fe^3+^ ions, the substantial pool of undegraded phytates is hypothesized to chelate the added ferric iron into electrochemically inactive complexes, thereby potentially masking the reducing potential of the matrix [66].

In contrast, WWG and WMs—both characterized by substantial initial phytic acid levels—showed a pronounced increase in FRAP at R7, reaching approximately 18–19 μmoL Fe(II)E/g d.b. This improvement is consistent with a substantial reduction in the phytate network during fermentation. Their rapid and intense acidification (final pH ~4.2) and high TTA values (~1.9% lactic acid) created optimal conditions for endogenous cereal phytases. The resulting phytic acid hydrolysis reduced iron chelation and “unmasked” the latent reducing capacity of the matrices, allowing ferric ions to fully participate in the FRAP redox reaction [28]. This process may have also contributed to the liberation of phenolics previously complexed within phytate–protein networks, potentially further enhancing the reducing power.

This dual action—phytic acid degradation and phenolic release—represents a highly plausible explanation for the synchronized rise in FRAP, DPPH, and ABTS observed in WWG and WMs at R7.

The addition of 20% semolina in all fermented S-series samples did not modify the antioxidant potential significantly with respect to their pure counterparts.

However, looking closely at the formulation matrix effects (Table 3), the incorporation of semolina acted as a crucial structural modulator rather than a simple inert diluent. In fiber-rich, highly resilient matrices like DWBS and WMS, the semolina provided a vital pool of readily fermentable carbohydrates. This nutritional synergy could have modified the physical architecture of the blends, potentially facilitating microbial penetration and improving the overall enzymatic release efficiency (RE%) of bound bioactives without penalizing the final antioxidant yield.

The kinetic profiles of pH and TTA (Figure 1) provide a coherent framework to interpret these results. In the scavenging assays, DWG reached the highest activity despite its high final pH (~4.8), indicating that radical-scavenging capacity depends primarily on the density of accumulated phenolic precursors rather than on the absolute intensity of acidification. Conversely, FRAP showed a strong dependence on acidification kinetics: WWG and WMs, which underwent the most vigorous fermentation, achieved the greatest recovery of reducing power, which appears closely linked to enhanced phytic acid degradation. Moreover, while single-strain or short-term fermentations commonly reported in the literature often reach an early plateau in antioxidant activity due to nutrient exhaustion and limited enzymatic release [67], our prolonged backslopping protocol maintained sustained microbial activity, as previously demonstrated in traditional sourdough ecosystems [37], thereby enabling a more extensive breakdown of matrix constraints.

Thus, while a moderate pH drop is sufficient to activate the scavenging potential of phenolic-rich matrices such as DWG, a more intense acidification—coupled with high TTA—is required to overcome mineral-chelation constraints in phytate-dense substrates. The robust TTA evolution in WMs further suggests sustained microbial metabolism and a broader enzymatic repertoire, resulting in the most balanced antioxidant profile across assays.

**Figure 8:**
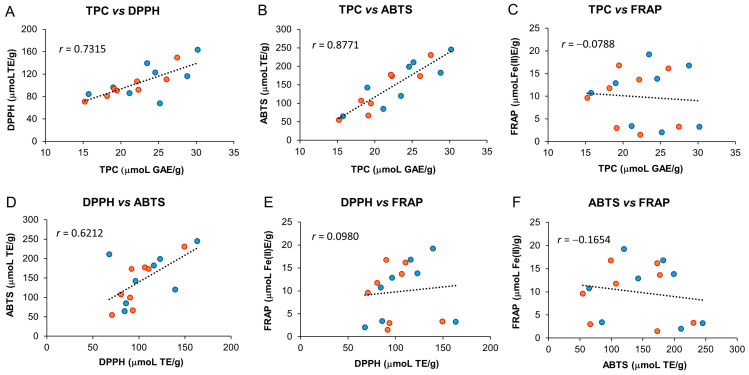
Scatter plots representing the Pearson correlation (*r*) between Total Phenolic Content (TPC) and antioxidant activities (DPPH, ABTS, and FRAP) (**A**–**C**), as well as inter-assay correlations (**D**–**F**), for T0 containing by-products alone (substrates) (in blue) and mixed with semolina (S-series) (in orange). Data include samples at T0 and after fermentation (R7). The dashed line indicates the linear regression fit. Units: TPC (μmoL GAE/g d.b.); DPPH and ABTS (μmoL TE/g d.b.); FRAP (μmoL Fe(II)/g d.b.).

A positive correlation was observed between TPC and radical-scavenging activities (Figure 8). In sourdough samples, Q-TPC showed a strong correlation with ABTS (*r* = 0.8771, *p* < 0.01, Panel B), while the correlation with DPPH was moderate (*r* = 0.7315, Panel A). This statistical trend is highly consistent with established literature [23,60,61], which widely documents that phenolic compounds released during bioprocessing act as effective radical scavengers; our findings thus strongly support the hypothesis that the observed increase in DPPH and ABTS antioxidant capacities is strongly associated with phenolic mobilization.

Conversely, no significant linear correlation was found between TPC and FRAP (r = −0.0788, Panel C), nor between FRAP and the radical-scavenging assays (DPPH vs. FRAP: r = 0.0980, Panel E; ABTS vs. FRAP: r = −0.1654, Panel F). This lack of association aligns with the “matrix-unmasking” hypothesis, suggesting that ferric-reducing power is likely governed by the phytase-mediated breakdown of iron–phytate complexes rather than the concentration of extractable phenolics. Because FRAP depends on the availability of free Fe^3+^ ions, its trajectory can be interpreted as an indirect reflection of phytic acid degradation dynamics rather than a direct measurement of specific biochemical pathways.

Overall, while DPPH and ABTS appear to primarily reflect the phenolic-driven antioxidant enhancement, FRAP is likely more responsive to the extent of phytic acid hydrolysis. These complementary chemical responses highlight the importance of integrating multiple assays to fully characterize the antioxidant potential of cereal by-products, serving as valuable indirect indicators of underlying matrix modifications rather than experimentally confirmed biochemical mechanisms [60].

**Figure 9:**
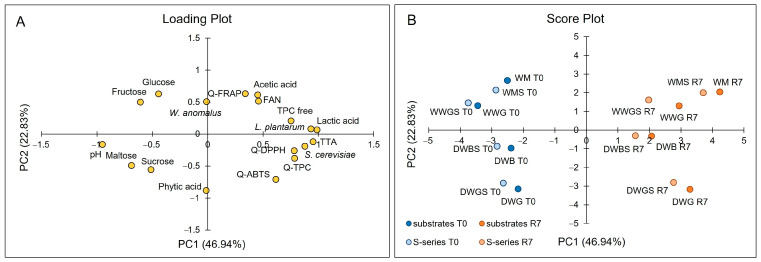
Principal Component Analysis (PCA) of the chemical, physical, and microbiological parameters during the fermentation process. (**A**) Loading plot showing the contribution and correlation of the monitored variables (pH, TTA, microbial counts, sugars, organic acids, phytic acid, TPC, and antioxidant activities) along the first two principal components (PC1 and PC2). (**B**) Score plot showing the distribution of the samples; blue symbols represent the initial time (T0), while orange symbols indicate the seven-refreshment stage (R7).

A Principal Component Analysis (PCA) was performed on the complete dataset to obtain an overall view of the fermentation process and to investigate the relationships among wheat milling by-products (WWG, DWG, DWB, WMs), semolina-containing formulations (S-series), and the monitored chemical, physical, and microbiological parameter variables at T0 and R7. The first two principal components explained 69.77% of the total variance (PC1 = 46.94%, PC2 = 22.83%), indicating a satisfactory representation of the system (Figure 9).

According to the loading plot (Figure 9A), PC1 was negatively associated with pH values and carbohydrates (glucose, fructose, sucrose, and maltose), which characterize the initial unfermented matrices. In contrast, PC1 was positively related to lactic acid, TTA, Free-TPC, antioxidant activity indices (Q-DPPH, Q-ABTS, Q-TPC), and microbial populations, including *L. plantarum* and *S. cerevisiae*. The opposite positioning of pH and acidification-related variables along PC1 reflects the expected decrease in pH associated with organic acid production during fermentation.

As shown in the score plot (Figure 9B), a clear separation of samples along PC1 was observed. Unfermented samples (T0, blue symbols) were located on the negative side of PC1, whereas all fermented samples (R7, orange symbols) shifted towards the positive side, indicating that PC1 mainly describes the progression of fermentation.

Substrates at T0 and R7 were dispersed along PC2, with no differences among them, prior and after stabilization, in carbohydrate availability.

The variable that contributed the most to the PC2 was phytic acid, which, together with acetic acid and FAN, highlighted specific metabolic traits (such as fermentative pathway activity and proteolysis) that differentiated the fermented wheat-based matrices WM and WWG series, located in the I and II quadrants, from the defatted by-products, located in the III and IV quadrants.

Interestingly, Q-FRAP was positioned separately from the other phenolic-related variables (Q-TPC, Q-DPPH, and Q-ABTS) along PC2, suggesting that FRAP values were not only related to the fermentation progression but might also be influenced by additional factors, such as acetic and phytic acid content.

The addition of semolina (S-series) had a negligible impact on the overall sample distribution, as S-samples closely clustered with their respective pure by-products and followed the exact same fermentation trajectory along PC1. This indicates that the inclusion of semolina does not alter the main biochemical and microbiological dynamics driven by the fermentation process.

Overall, PCA reveals that, despite initial compositional differences among the raw by-products, fermentation drives a highly consistent shift toward acidification, microbial growth, and biochemical modification. The spatial proximity of the fermented samples suggests that this bioprocess narrows the functional gaps between different substrates, yielding a matrix with a more uniform functional profile that holds promising potential for future industrial scaling.

## 3. Materials and Methods

### 3.1. By-Products, Chemicals and Reagents

Defatted durum wheat germ (DWG), defatted durum wheat bran (DWB) and remilled semolina (S) were kindly provided by Casillo Next Gen Food (Corato, Bari, Italy). Whole wheat germ (WWG) was provided by Molino Agostini (Massignano, Italy) and durum wheat middlings (WMs) by Molino F.lli Brundu (Macomer, Italy).

2,4,6-trinitrobenzenesulfonic acid (TNBS) (5% solution), Folin–Ciocalteu’s reagent, sodium carbonate (Na_2_CO_3_), iron chloride (III) hexahydrate (FeCl_3_ × 6 H_2_O), iron sulphate (II) heptahydrate (Fe_2_SO_4_ × 7 H_2_O), Trolox, Gallic acid, 2,2-diphenyl-1-picryl hydrazyl (DPPH), 2,4,6-Tri(2-pyridyl)-S-triazine (TPTZ), 2,20-azinobis (3-ethylbenzothiazoline-6-sulphonic acid-) diammonium salt (ABTS), sodium acetate trihydrated (NaC_2_H_3_O_2_ × 3 H_2_O), acetic acid (CH_3_COOH) and potassium persulfate (K_2_O_8_S_2_) were obtained from Sigma-Aldrich (St. Louis, MO, USA). All chemicals and solvents were of analytical grade and used without further purification.

### 3.2. Characterization of Raw Wheat By-Products

The moisture and ash contents of the raw wheat by-products (WWG, DWG, DWB, and WMs) were determined at 130 °C and 580 °C, respectively, until a constant weight was reached, using a Thermostep Thermogravimetric Analyzer (Eltra GmbH, Haan, Germany). The protein content (N × 5.7) was quantified according to AACC combustion method 46–30 using a Rapid N Cube analyzer (Elementar Analysensysteme GmbH, Langenselbold, Germany) [68]. Total dietary fiber content was measured using a Total Dietary Fiber assay kit (Megazyme, Wicklow, Ireland). The total lipid content was determined following the Folch extraction method [69], with the modifications described by Melis et al. [70].

The water binding capacity (WBC) of the raw by-products was determined according to De La Hera et al. [71] with minor modifications: 1 g of sample was mixed with 10 mL of distilled water, allowed to stand at room temperature for 24 h, and then centrifuged at 2000× *g* for 15 min. The supernatant was subsequently removed, and the remaining pellet was weighed.

The endogenous microbiota of the raw by-products was evaluated by suspending 10 g of each sample in 90 mL of a sterile peptone solution (1 g/L of peptone in distilled water). After homogenization in a Stomacher blender for 2 min, serial decimal dilutions were spread on Plate Count Agar (PCA; Oxoid, Basingstoke, Hampshire, UK) to determine the total aerobic bacteria, and on YEPD Agar (yeast extract 10 g/L, peptone 10 g/L, glucose 20 g/L, agar 17 g/L, pH 5.5) to enumerate yeasts and molds. Plates were incubated at 30 °C for 48 h. In the raw WWG and WMs, the total aerobic bacterial count was approximately 5 Log_10_ CFU g^−1^, while the yeast and mold counts were around 2 Log_10_ CFU g^−1^. Conversely, in the raw DWG and DWB, the total bacterial count was below 2 Log_10_ CFU g^−1^, whereas the yeast and mold counts were approximately 4 Log_10_ CFU g^−1^.

### 3.3. Sourdough Production and Propagation

The sourdough substrates (designated as WWG, DWG, DWB, and WMs) were prepared by mixing sterile water with wheat by-products according to their water binding capacity. An additional set of four substrates (WWGS, DWGS, DWBS, and WMS) was prepared by blending each by-product with semolina, at an 8:2 ratio (*w*/*w*). Water was added according to the hydration level required for each by-product, while semolina was hydrated at a 1:1 ratio (*w*/*w*). The detailed composition and dough yield of each substrate are reported in Table 3.

Starter cultures of *L. plantarum* PCC2397, *S. cerevisiae* PCC1602, and *W. anomalus* PCC1629, belonging to the Microbial Culture Collection of Porto Conte Ricerche and previously selected for their metabolic properties, were used as an inoculum. Strains were propagated as already described by Fois et al. [72]. Sourdough fermentation was performed at 25 °C for 8 h in a bioreactor (AFTL5, SITEP S.r.l., Voghiera, Italy), after which the sourdoughs were cooled to 5 °C until the next fermentation step.

**Figure 10:**
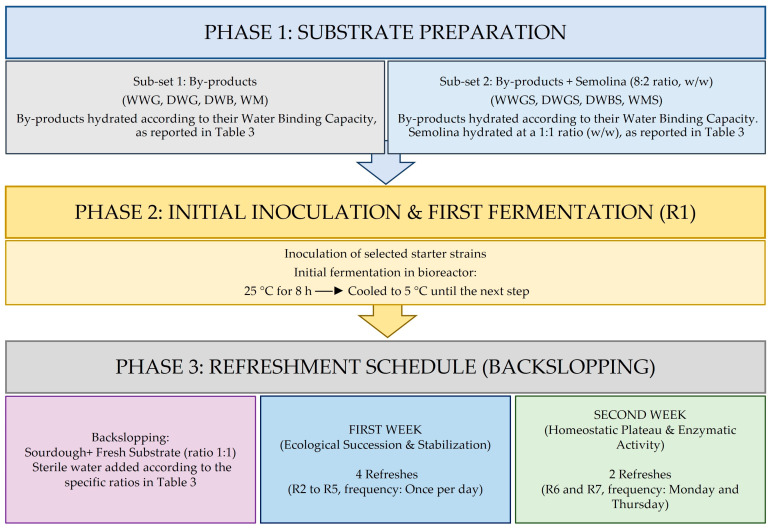
Flowchart of the sourdough production workflow, illustrating the substrate preparation (pure by-products vs. semolina blends), initial inoculation, and chronological two-week backslopping schedule (R1–R7).

Refreshments were performed by backslopping for two weeks, by mixing a leftover portion of sourdough with the same amount of fresh by-products or a semolina blend (at a ratio of 1:1) [72]. Sterile water was added according to the ratios reported in Table 3. After inoculation and the first fermentation step (R1), the sourdoughs were refreshed four times during the first week (R1-R5, once per day), in order to obtain a stable and mature sourdough, and twice during the second week (R6 and R7, Monday and Thursday). The sourdough production workflow is illustrated in Figure 10.

### 3.4. Sourdough Analyses

Microbial count, total titratable acidity (TTA) and pH were determined on refrigerated sourdough samples, according to Fois et al. [72]. TTA values were expressed in % lactic acid.

Phytic acid content was determined on freeze-dried samples using the Phytic Acid (Phytate)/Total Phosphorus assay kit (Megazyme, Wicklow, Ireland).

To rule out the possibility that the industrial oil extraction process had denatured the endogenous phytases present in defatted wheat germ (DWG) and defatted wheat bran (DWB), an enzymatic assay was conducted under optimal conditions according to Peers [73], with some modifications. Briefly, 5 g of each defatted substrate was suspended in a 0.25 M sodium acetate buffer (pH 5.2) and incubated at 45 °C for 3 h. The first experiment was conducted using a substrate-to-buffer ratio of 1:10 (*w*/*v*). In a second experiment, the hydration conditions used during fermentation were replicated, and the substrate-to-buffer ratio was adjusted to 1:2 for DWG and 1:2.5 for DWB. In both experiments, the phytic acid content was measured at the end of the incubation period and compared with the baseline values at T0.

Carbohydrates and organic acids in sourdough were determined as previously reported [72], following the manufacturer’s instructions. Briefly, 5.0 g of freeze-dried dough was dispersed in 45 mL of Milli-Q water, stirred for 30 min, and centrifuged at 15,000× *g* for 10 min at 20 °C.

Extracts were stored at −80 °C until analysis. Metabolites were quantified using Enzytec^TM^ kits (R-Biopharm AG, Darmstadt, Germany) on an iCubio i-Magic M9 analyzer (Origlia S.r.L, Italy). The results were expressed as %*w*/*w*. All extractions and analyses were performed in duplicate.

FAN was quantified according to described protocols [74,75], with slight modifications. Samples (0.2 mL) were mixed with 1 mL of borate buffer (0.1 M, pH 10) and 1 mL of TNBS solution (0.01%), incubated at 42 °C for 10 min in the dark, and read at 340 nm. The results were expressed as mg of leucine equivalents per g of dough (mg Leu E/g). Analyses were performed in duplicate.

### 3.5. Determination of Phenolic Content

#### 3.5.1. Sample Preparation

Samples were freeze-dried and consecutively passed through a sieve having a mesh size of 45 (355 μm) to produce a fine powder. Samples were stored at 4 °C until analysis.

#### 3.5.2. Determination of Free Phenolic Content

Conventional solid–liquid extraction of free phenolic content (Free-TPC) was performed as previously reported [54,55], with slight modifications: 0.1 g of the freeze-dried samples were magnetically stirred for 60 min at 25 °C with 2 mL of ethanol/H_2_O (50:50, *v*/*v*). The dispersions were then centrifuged at 14,000 rpm for 5 min at 15 °C (Centrifuge 5430 R model, Eppendorf AG, Hamburg, Germany). For the colorimetric assay, 100 μL of the resulting supernatant was reacted with 2 mL of Folin–Ciocalteu reagent diluted (1:10) in ethanol/H_2_O (50:50, *v*/*v*) for 5 min. Following this, 2 mL of 7% Na_2_CO_3_ was added. The samples were incubated in the dark for 60 min at room temperature. After centrifugation, the absorbance was measured at 760 nm using an Agilent Cary 60 spectrophotometer (Agilent, Santa Clara, CA, USA). Results were expressed as micromoles of gallic acid equivalents (GAE) per gram of product (μmoL GAE/g) based on a standard calibration curve. GAE is used as a conventional benchmarking standard. Extractions and analyses were performed in duplicate.

#### 3.5.3. Q-TPC Assay

The total phenolic content (Q-TPC) was analyzed by using the Q-FC, according to the protocol described by Del Pino-García et al. [76], with slight modifications. Briefly, 10 ± 0.1 mg of the freeze-dried sample was accurately weighed into an amber glass vial. To facilitate the surface-solid reaction, the sample was mixed with 2 mL of Folin–Ciocalteu reagent diluted (1:10, *v*/*v*) in ethanol/H_2_O (50:50 *v*/*v*), and the mixture was magnetically stirred for 10 min. Subsequently, 2 mL of Na_2_CO_3_ solution (7%, *w*/*v*) was added. The reaction mixture was then kept under constant stirring for 60 min at room temperature in the dark to ensure complete incubation. After centrifugation at 14,000 rpm for 5 min at 15 °C, the absorbance of the supernatant was measured at 750 nm using an Agilent Cary 60 spectrophotometer (Agilent, Santa Clara, CA, USA). The Q-TPC values were expressed as micromoles of gallic acid equivalents per gram of dry basis product (μmol GAE/g d.b.) by means of a standard dose–response curve. All extractions and analyses were performed in duplicate.

It is important to underline that, with both the Folin–Ciocalteu and FRAP methods being unspecific methods, part of the reported measurements might be due to interfering compounds. Specifically, a Folin–Ciocalteu reagent not only measures the number of phenolic compounds but may also react with any reducing substance. It, therefore, measures the total reducing capacity of a sample.

To evaluate the efficacy of the fermentation process in mobilizing bound phenolic compounds, the release efficiency (RE%) was calculated for each substrate. RE represents the percentage of bound phenolic compounds initially present in the matrix (T0) that are converted into free (bioaccessible) forms after fermentation (R7).

The RE was determined using the following equation:

RE (%) = (Free R7 − Free T0)/Bound T0 × 100

where Free R7 is the concentration of free phenolic compounds (μmoL GAE/g d.b.) after seven refreshments (R7), Free T0 is the initial concentration of free phenolic compounds, and Bound T0 represents the initial concentration of bound phenolic content, calculated as the difference between total and free phenolics (Bound T0 = Q-TPC − Free-TPC T0) in the unfermented matrix.

This approach allows for a standardized comparison of the enzymatic deconstruction of the different matrices (WWG, DWG, DWB, and WMs), independent of their initial phenolic content. Positive RE values indicate a net release of phenolics from the insoluble matrix, whereas negative values suggest that degradation or transformation processes prevail over phenolic mobilization.

### 3.6. Determination of Antioxidant Activity by QUENCHER Method

#### 3.6.1. Q-DPPH Assay

The DPPH radical-scavenging capacity (Q-DPPH) was determined according to the protocol previously described [54]. Initially, DPPH (~40 mg/L) was dissolved in absolute ethanol and subsequently diluted with water to achieve a 50:50 (*v*/*v*) ethanol/water ratio. The final concentration of the radical solution was adjusted to reach an absorbance between 0.750 and 0.800 AU at a wavelength of 525 nm. Then, 10 ± 0.1 mg of the freeze-dried sample was accurately weighed into amber glass vials and mixed with 10 mL of the DPPH^•^ working solution. The mixture was vortexed for 1 min and then kept under continuous magnetic stirring for 60 min at room temperature in the dark to facilitate the surface-solid scavenging reaction. Following incubation, the samples were centrifuged at 14,000 rpm for 5 min. The absorbance of the clear supernatant was measured at 525 nm. The radical-scavenging activity was calculated as decolorization (i.e., the loss in absorbance) by subtracting the sample absorbance from that of the initial DPPH^•^ working solution. Trolox was used as the standard to perform a calibration curve, and the results were expressed as micromoles of Trolox equivalents per gram of dry basis product (μmoL TE/g d.b.). All analyses were performed in duplicate.

#### 3.6.2. Q-ABTS Assay

The ABTS^•+^ radical-scavenging capacity (Q-ABTS) was evaluated according to previously reported methods [77,78], with some modifications. The ABTS^•+^ stock solution was prepared by reacting 1.0 mL of a sodium persulfate stock solution (6.89 × 10^−3^ M) with 99.0 mL of an ABTS stock solution (5.0 × 10^−4^ M). This mixture was kept in the dark at room temperature for 12–16 h to allow for complete radical stabilization. On the day of analysis, the working solution was prepared by diluting the stock solution in an ethanol/water mixture (50:50 *v*/*v*), to achieve an initial absorbance between 0.750 and 0.800 AU at 734 nm. Then, 10 ± 0.1 mg of the sample was accurately weighed into amber glass vials, and the reaction was initiated by adding 10 mL of ABTS^•+^ working solution. The mixture was immediately vortexed for 1 min and then placed under continuous magnetic stirring for 30 min at room temperature in the dark to facilitate the surface-solid reaction. Following incubation, the samples were centrifuged at 14,000 rpm for 5 min to obtain a clear supernatant. The absorbance was measured at 734 nm. The radical-scavenging activity was calculated as decolorization by subtracting the sample absorbance from that of the initial ABTS^•+^ working solution. Trolox was used as the standard to construct a linear calibration curve, and the results were expressed as micromoles of Trolox equivalents per gram of dry basis product (μmoL TE/g d.b.). All analyses were performed in duplicate.

#### 3.6.3. Q-FRAP Assay

The ferric-reducing antioxidant power (Q-FRAP) was determined using an extraction-free approach adapted from the method previously described [76,79]. The FRAP working reagent was freshly prepared by mixing a 10 mM TPTZ solution and a 20 mM FeCl_3_ × 6H_2_O solution in a 300 mM sodium acetate buffer (pH 3.6) at a volume ratio of 1:1:10 (*v*/*v*/*v*). This mixture was subsequently diluted 1:10 (*v*/*v*) in water. Briefly, 10 ± 0.1 mg of the freeze-dried sample was accurately weighed into amber glass vials, and the reaction was initiated by adding 10 mL of the diluted FRAP reagent. The mixture was kept under continuous magnetic stirring for 60 min in the dark to facilitate the surface-solid reduction process. Following incubation, the samples were immediately centrifuged at 14,000 rpm for 5 min to obtain a clear supernatant. The reduction in the colorless Fe(III)-tripyridyltriazine complex to the intensely blue Fe(II) form was monitored by measuring the increase in absorbance at 593 nm against a reagent blank. The results were expressed as micromoles of Fe(II) equivalents per gram of dry basis product (μmoL Fe(II)E/g d.b.) by means of a linear calibration curve obtained using different concentrations of FeSO_4_ × 7H_2_O. All analyses were performed in duplicate.

### 3.7. Statistical Analyses

To evaluate the effects of raw materials and the fermentation process, data were subjected to Two-Way Analysis of Variance (ANOVA). The statistical model included ‘Substrate formulation’ (8 levels: WWG, DWG, DWB, and WMs, both pure and blended with 20% semolina) and ‘Fermentation time’ (2 levels: T0 and R7) as fixed factors.

For pH and TTA, analyses were performed in triplicate directly on the matrix. For the antioxidant assays, to account for experimental and extraction variance, two entirely independent chemical extractions (from separate sample weightings) were performed for each matrix profile at each timepoint (n = 2 experimental replicates), with instrumental readings performed in duplicate. To ensure statistical consistency, all technical and analytical replicates were averaged per batch prior to statistical modeling, ensuring that the true independent replication served as the unit of analysis in the ANOVA.

Prior to the ANOVA, the parametric assumptions of normality and homoscedasticity were verified using the Shapiro–Wilk test and Levene’s test, respectively.

The Bonferroni post hoc test was applied for multiple comparisons to resolve significant differences (*p* < 0.05) between timepoints and formulations.

Subsequently, a Principal Component Analysis (PCA) was performed on the normalized dataset using a correlation matrix. The selection of the optimal number of principal components (PCs) was based on the Kaiser criterion (eigenvalues > 1) and the cumulative explained variance. The results were visualized through a two-dimensional loading plot, illustrating the contribution and correlation of the measured variables (microbial counts, sugars, organic acids, and antioxidant activities) along the principal components, and a score plot, describing samples’ distribution. All univariate and multivariate statistical analyses, as well as graphical representations, were performed using the XLSTAT Essentials software (version 2026.1.0, Addinsoft, Paris, France). Statistical significance was set at *p* < 0.05.

## 4. Conclusions

This study showed that long-term sourdough fermentation is an effective strategy for the valorization of diverse wheat milling by-products. By integrating conventional extraction with the solid-state QUENCHER approach, we demonstrated that traditional liquid assays substantially underestimate the antioxidant potential of fiber-bound phenolics by 40–60%. Fermentation enhanced both phenolic release and phytic acid degradation, suggesting the involvement of two complementary antioxidant pathways.

Among the investigated substrates, whole wheat germ and wheat middlings exhibited the most pronounced functional response, achieving a phytic acid reduction of up to 60%, a high FAN accumulation (up to 2.6 mg/g d.b.), and a marked boost in ferric-reducing antioxidant power (FRAP up to 18–19 µmol Fe(II)E/g d.b.). Conversely, defatted matrices showed structural limitations linked to industrial processing (e.g., hexane treatment), which restricted enzymatic mobilization. PCA further supported that fermentation drives all matrices toward a convergent functional profile despite their initial compositional differences. Overall, microbial bioprocessing through controlled sourdough fermentation provides a promising strategy to transform cereal by-products into high-value, bioactive, and nutrient-dense ingredients.

Despite these promising functional outcomes, a primary limitation of this study is that fermentation dynamics were evaluated strictly under laboratory-scale conditions, without assessing the rheological, technological, and sensory impacts on final food products. Therefore, future research prospects should focus on: (i) performing real-world baking trials with long-term sourdough, to evaluate nutritional properties, shelf life and consumer acceptability of bread; (ii) investigating physical or enzymatic pre-treatments (e.g., micronization or exogenous enzymes) to overcome the structural resilience of defatted matrices; and (iii) conducting a life-cycle assessment to validate the economic and environmental viability of scaling up this bioprocess for industrial applications.

## Figures and Tables

**Table 1:** Wheat by-product characterization.

By-Product	Moisture Content (%)	Ash (%) ^1^	Protein Content (%) ^1^	Lipids (%) ^1^	Total Dietary Fiber (%) ^1^
Whole wheat germ (WWG)	12.31 ± 0.09	3.09 ± 0.01	15.50 ± 0.13	4.41 ± 0.06	34.91 ± 0.40
Defatted durum wheat germ (DWG)	7.87 ± 0.04	9.30 ± 0.05	25.27 ± 0.05	2.34 ± 0.04	24.38 ± 0.04
Defatted durum wheat bran (DWB)	7.45 ± 0.02	4.81 ± 0.03	17.52 ± 0.09	1.15 ± 0.03	39.28 ± 0.04
Wheat middlings (WMs)	9.04 ± 0.01	5.87 ± 0.06	18.80 ± 0.02	6.42 ± 0.03	46.60 ± 1.06

(%) ^1^ values referred to d.b. per dry basis.

**Table 2:** Release efficiency (RE%) of phenolic compounds after seven refreshments (R7). RE (%) expresses the percentage of the initial bound reservoir converted into free forms after fermentation, calculated as RE% = [(Free R7 − Free T0)/Bound T0] × 100. Bound T0 was calculated as the difference between total and free phenolics (Q-TPC T0 − Free T0). Data variability is shown in Figure 6, where results are presented as mean ± SD.

Sample	Free T0 (μmoL GAE/g d.b.)	Free R7 (μmoL GAE/g d.b.)	Q-TPC T0 (μmoL GAE/g d.b.)	Bound T0 (μmoL GAE/g d.b.)	RE (%)
WWG	9.11	14.34	15.74	6.63	78.9
DWG	12.96	13.90	25.17	12.21	7.7
DWB	10.91	10.69	19.00	8.09	−2.7
WMs	13.37	17.61	21.15	7.78	54.5
WWGS	8.48	13.80	15.23	6.75	78.8
DWGS	11.27	12.57	22.33	11.06	11.8
DWBS	10.31	11.91	18.20	7.89	20.3
WMS	11.66	17.32	19.16	7.50	75.5

**Table 3:** Composition of sourdough substrates.

Sourdough Sample	By-Product (g)	Semolina (g)	Total Water (g)	Water:By-Product Ratio (*w*/*w*)	Dough Yield
WWG	840	-	2352	2.8:1	380
WWGS	756	189	2306	2.8:1	344
DWG	1100	-	2200	2:1	300
DWGS	920	230	2070	1:2	280
DWB	900	-	2250	2.5:1	350
DWBS	800	200	2200	2.5:1	320
WMs	800	-	2400	3:1	400
WMS	720	180	2340	3:1	360

## Data Availability

The data presented in this study are available upon request from the corresponding author.

## Abbreviations

The following abbreviations are used in this manuscript:

WWG	Whole wheat germ
DWG	Defatted wheat germ
DWB	Defatted wheat bran
WMs	Wheat middlings
TTA	Total titratable acidity
FAN	Free amino nitrogen
TNBS	2,4,6-trinitrobenzenesulfonic acid
TPC	Total phenolic content
QUENCHER	Quick, Easy, New, Cheap, and High-throughput Extraction-free
DPPH	2,2-diphenyl-1-picryl hydrazyl
ABTS	2,20-azinobis (3-ethylbenzothiazoline-6-sulphonic acid-) diammonium salt
FRAP	Ferric-reducing/antioxidant power
PCA	Principal Component Analysis

## References

[1] Deyalage S.T., House J.D., Thandapilly S.J., Malalgoda M. (2024). Nutritional characteristics and physicochemical properties of ancient wheat species for food applications. Food Biosci..

[2] Danciu C.A., Tulbure A., Stanciu M.A., Antonie I., Capatana C., Zerbeș M.V., Giurea R., Rada E.C. (2023). Overview of the Sustainable Valorization of Using Waste and By-Products in Grain Processing. Foods.

[3] Khashaba R.A., Lou H., Li Y., Saeed Omer S.H., Wang X., Gu Z., Zhao R. (2026). Optimizing Wheat Milling By-Products: An Overview of Processing Techniques. Foods.

[4] Skendi A., Zinoviadou K.G., Papageorgiou M., Rocha J.M. (2020). Advances on the Valorisation and Functionalization of By-Products and Wastes from Cereal-Based Processing Industry. Foods.

[5] Food and Agriculture Organization of the United Nations FAO Cereal Supply and Demand Brief. World Food Situation. https://www.fao.org/worldfoodsituation/csdb/en/.

[6] Islam M.Z., Zheng L. (2025). Why is it necessary to integrate circular economy practices for agri-food sustainability from a global perspective?. Sustain. Dev..

[7] Chen Z., Mense A.L., Brewer L.R., Shi Y.C. (2024). Wheat bran layers: Composition, structure, fractionation, and potential uses in foods. Crit. Rev. Food Sci. Nutr..

[8] Călinoiu L.F., Vodnar D.C. (2018). Whole Grains and Phenolic Acids: A Review on Bioactivity, Functionality, Health Benefits and Bioavailability. Nutrients.

[9] Ma D., Wang C., Feng J., Xu B. (2021). Wheat grain phenolics: A review on composition, bioactivity, and influencing factors. J. Sci. Food Agric..

[10] Ribeiro da Silva Lima L., Barros Santos M.C., Gomes P.W.P., Fernández-Ochoa Á., Simões Larraz Ferreira M. (2024). Overview of the metabolite composition and antioxidant capacity of seven major and minor cereal crops and their milling fractions. J. Agric. Food Chem..

[11] Zhu Y., Sang S. (2017). Phytochemicals in whole grain wheat and their health-promoting effects. Mol. Nutr. Food Res..

[12] Rudrapal M., Khairnar S.J., Khan J., Dukhyil A.B., Ansari M.A., Alomary M.N., Alshabrmi F.M., Palai S., Deb P.K., Devi R. (2022). Dietary polyphenols and their role in oxidative stress-induced human diseases: Insights into protective effects, antioxidant potentials and mechanism(s) of action. Front. Pharmacol..

[13] Gasmi A., Mujawdiya P.K., Noor S., Lysiuk R., Darmohray R., Piscopo S., Lenchyk L., Antonyak H., Dehtiarova K., Shanaida M. (2022). Polyphenols in Metabolic Diseases. Molecules.

[14] Brandolini A., Hidalgo A. (2012). Wheat germ: Not only a by-product. Int. J. Food Sci. Nutr..

[15] Marzocchi S., Caboni M.F., Greco Miani M., Pasini F. (2022). Wheat Germ and Lipid Oxidation: An Open Issue. Foods.

[16] Ariyarathna P., Mizera P., Walkowiak J., Dziedzic K. (2025). Physicochemical and Functional Properties of Soluble and Insoluble Dietary Fibers in Whole Grains and Their Health Benefits. Foods.

[17] Zhu K.X., Lian C.X., Guo X.N., Peng W., Zhou H.M. (2011). Antioxidant activities and total phenolic contents of various extracts from defatted wheat germ. Food Chem..

[18] Liu F., Chen Z., Shao J., Wang C., Zhan C. (2017). Effect of fermentation on the peptide content, phenolics and antioxidant activity of defatted wheat germ. Food Biosci..

[19] Fărcaș A., Drețcanu G., Pop T.D., Enaru B., Socaci S., Diaconeasa Z. (2021). Cereal processing by-products as rich sources of phenolic compounds and their potential bioactivities. Nutrients.

[20] Ed Nignpense B., Francis N., Blanchard C., Santhakumar A.B. (2021). Bioaccessibility and Bioactivity of Cereal Polyphenols: A Review. Foods.

[21] Bas T.G. (2026). Dietary Polyphenols (Flavonoids) Derived from Plants for Use in Therapeutic Health: Antioxidant Performance, ROS, Molecular Mechanisms, and Bioavailability Limitations. Int. J. Mol. Sci..

[22] Ram S., Narwal S., Gupta O.P., Pandey V., Singh G.P., Om Prakash G. (2020). Anti-nutritional factors and bioavailability: Approaches, challenges, and opportunities. Wheat and Barley Grain Biofortification.

[23] Verni M., Rizzello C.G., Coda R. (2019). Fermentation biotechnology applied to cereal industry by-products: Nutritional and functional insights. Front. Nutr..

[24] Reque P.M., Pinilla C.M.B., Tinello F., Corich V., Lante A., Giacomini A., Brandelli A. (2020). Biochemical and functional properties of wheat middlings bioprocessed by lactic acid bacteria. J. Food Biochem..

[25] Islam S., Miah A.S., Islam F., Tisa K.J., Bhuiyan H.R., Bhuiyan M.N.I., Afrin S., Ahmed K.S., Hossain H. (2024). Fermentation with lactic acid bacteria enhances the bioavailability of bioactive compounds of whole wheat flour. Appl. Food Res..

[26] De Vuyst L., Comasio A., Van Kerrebroeck S. (2023). Sourdough production: Fermentation strategies, microbial ecology, and use of non-flour ingredients. Crit. Rev. Food Sci. Nutr..

[27] Kim K.H., Tsao R., Yang R., Cui S.W. (2006). Phenolic acid profiles and antioxidant activities of wheat bran extracts and the effect of hydrolysis conditions. Food Chem..

[28] Sadowska-Bartosz I., Bartosz G. (2022). Evaluation of The Antioxidant Capacity of Food Products: Methods, Applications and Limitations. Processes.

[29] Gökmen V., Serpen A., Fogliano V. (2009). Direct measurement of the total antioxidant capacity of foods: The ‘QUENCHER’ approach. Trends Food Sci. Technol..

[30] Cömert E.D., Gökmen V. (2017). Antioxidants bound to an insoluble food matrix: Their analysis, regeneration behavior, and physiological importance. Compr. Rev. Food Sci. Food Saf..

[31] King D.L., Jasthi B., Pettit J., Wrigley C., Batey I., Miskelly D. (2017). Appendix 1—Composition of Grains and Grain Products. Cereal Grains.

[32] Gänzle M.G. (2014). Enzymatic and bacterial conversions during sourdough fermentation. Food Microbiol..

[33] Katina K., Arendt E., Liukkonen K.H., Autio K., Flander L., Poutanen K. (2005). Potential of sourdough for healthier cereal products. Trends Food Sci. Technol..

[34] Rizzello C.G., Nionelli L., Coda R., De Angelis M., Gobbetti M. (2010). Effect of sourdough fermentation on stabilisation, and chemical and nutritional characteristics of wheat germ. Food Chem..

[35] Fernández-Peláez J., Paesani C., Gómez M. (2020). Sourdough Technology as a Tool for the Development of Healthier Grain-Based Products: An Update. Agronomy.

[36] Dobre A.A., Cucu E.M., Belc N. (2024). Influence of Technological Parameters on Sourdough Starter Obtained from Different Flours. Appl. Sci..

[37] Minervini F., De Angelis M., Di Cagno R., Gobbetti M. (2014). Ecological parameters influencing microbial diversity and stability of traditional sourdough. Int. J. Food Microbiol..

[38] Coda R., Cassone A., Rizzello C.G., Nionelli L., Cardinali G., Gobbetti M. (2011). Antifungal activity of *Wickerhamomyces anomalus* and *Lactobacillus plantarum* during sourdough fermentation: Identification of novel compounds and long-term effect during storage of wheat bread. Appl. Environ. Microbiol..

[39] Magallanes Lopez A.M., Simsek S. (2021). Pathogens control on wheat and wheat flour: A review. Cereal Chem..

[40] Shivaprasad D.P., Rivera J., Siliveru K. (2024). Acidic water tempering and heat treatment, a hurdle approach to reduce wheat Salmonella load during tempering and its effects on flour quality. Food Res. Int..

[41] Loponen J., Mikola M., Katina K., Sontag-Strohm T., Salovaara H. (2004). Degradation of HMW glutenins during wheat sourdough fermentations. Cereal Chem..

[42] Gänzle M.G., Vermeulen N., Vogel R.F. (2007). Carbohydrate, peptide and lipid metabolism of lactic acid bacteria in sourdough. Food Microbiol..

[43] Wang H., Johnson L.A., Wang T. (2004). Preparation of soy protein concentrate and isolate from extruded-expelled soybean meals. J. Am. Oil Chem. Soc..

[44] Meriles S.P., Steffolani M.E., León A.E., Penci M.C., Ribotta P.D. (2019). Physico-chemical characterization of protein fraction from stabilized wheat germ. Food Sci. Biotechnol..

[45] Rousta N., Taherzadeh M.J. (2026). Measuring phytic acid in complex and fermented food matrices: Modified protocol using enzymatic analysis. JSFA Rep..

[46] Gómez M., González J., Oliete B. (2012). Effect of extruded wheat germ on dough rheology and bread quality. Food Bioprocess Technol..

[47] Zhu K.X., Sun X.H., Chen Z.C., Peng W., Qian H.F., Zhou H.M. (2010). Comparison of functional properties and secondary structures of defatted wheat germ proteins separated by reverse micelles and alkaline extraction and isoelectric precipitation. Food Chem..

[48] Bohn L., Meyer A.S., Rasmussen S.K. (2008). Phytate: Impact on environment and human nutrition. A challenge for molecular breeding. J. Zhejiang Univ. Sci. B.

[49] Cheryan M., Rackis J.J. (1980). Phytic acid interactions in food systems. Crit. Rev. Food Sci. Nutr..

[50] Rezaei K., Jenab E., Temelli F. (2007). Effects of Water on Enzyme Performance with an Emphasis on the Reactions in Super-critical Fluids. Crit. Rev. Biotechnol..

[51] McKie V.A., McCleary B.V. (2016). A novel and rapid colorimetric method for measuring total phosphorus and phytic acid in foods and animal feeds. J. AOAC Int..

[52] Bayat E., Moosavi-Nasab M., Fazaeli M., Majdinasab M., Mirzapour-Kouhdasht A., Garcia-Vaquero M. (2022). Wheat Germ Fermentation with *Saccharomyces cerevisiae* and *Lactobacillus plantarum*: Process Optimization for Enhanced Composition and Antioxidant Properties In Vitro. Foods.

[53] Kumar A., Saranyadevi S., Thirumalaisamy S.K., Dapana Durage T.T., Jaiswal S.G., Kavitake D., Wei S. (2025). Phenolic acids in fermented foods: Microbial biotransformation, antioxidant mechanisms, and functional health implications. Front. Mol. Biosci..

[54] Tufan A.N., Çelik S.E., Özyürek M., Güçlü K., Apak R. (2013). Direct measurement of total antioxidant capacity of cereals: QUENCHER-CUPRAC method. Talanta.

[55] Serpen A., Gökmen V., Fogliano V. (2012). Solvent effects on total antioxidant capacity of foods measured by direct QUENCHER procedure. J. Food Compos. Anal..

[56] Serpen A., Gökmen V., Pellegrini N., Fogliano V. (2008). Direct measurement of the total antioxidant capacity of cereal products. J. Cereal Sci..

[57] Çelik E.E., Cömert E.D., Gökmen V. (2024). The power of the QUENCHER method in measuring total antioxidant capacity of foods: Importance of interactions between different forms of antioxidants. Talanta.

[58] Munteanu I.G., Apetrei C. (2021). Analytical Methods Used in Determining Antioxidant Activity: A Review. Int. J. Mol. Sci..

[59] Bibi Sadeer N., Montesano D., Albrizio S., Zengin G., Mahomoodally M.F. (2020). The Versatility of Antioxidant Assays in Food Science and Safety—Chemistry, Applications, Strengths, and Limitations. Antioxidants.

[60] Adil M.Z., Oztekin S., Aziz A., Gunal-Koroglu D., Capanoglu E., Moreno A., Khalid W., Esatbeyoglu T. (2026). Fermentation-Based Valorization of Agro-Industrial Cereal Waste and By-Products. Trends Food Sci. Technol..

[61] Zhang J., Liu M., Zhao Y., Zhu Y., Bai J., Fan S., Zhu L., Song C., Xiao X. (2022). Recent Developments in Fermented Cereals on Nutritional Constituents and Potential Health Benefits. Foods.

[62] Teslić N., Bojanić N., Rakić D., Takači A., Zeković Z., Fišteš A., Bodroža-Solarov M., Pavlić B. (2019). Defatted wheat germ as source of polyphenols—Optimization of microwave-assisted ex-traction by RSM and ANN approach. Chem. Eng. Process. Process Intensif..

[63] Arnao M.B., Cano A., Acosta M. (2001). The hydrophilic and lipophilic contribution to total antioxidant activity. Food Chem..

[64] Nenadis N., Wang L.F., Tsimidou M., Zhang H.Y. (2004). Estimation of scavenging activity of phenolic compounds using the ABTS^•+^ assay. J. Agric. Food Chem..

[65] Du K., Tian S., Chen H., Gao S., Dong X., Yan F. (2022). Application of enzymes in the preparation of wheat germ polypeptides and their biological activities. Front. Nutr..

[66] Graf E., Empson K.L., Eaton J.W. (1987). Phytic acid. A natural antioxidant. J. Biol. Chem..

[67] Verni M., Verardo V., Rizzello C.G. (2019). How Fermentation Affects the Antioxidant Properties of Cereals and Legumes. Foods.

[68] AACC (2010). Approved Methods of Analysis.

[69] Folch J., Lees M., Stanley G.H.S. (1957). A simple method for the isolation and purification of total lipids from animal tissues. J. Biol. Chem..

[70] Melis R., Vitangeli I., Anedda R. (2022). Effect of fish diet and cooking mode on the composition and microstructure of ready-to-eat fish fillets of gilthead sea bream (*Sparus aurata*) juveniles. J. Food Compos. Anal..

[71] De La Hera E., Gomez M., Rosell C.M. (2013). Particle size distribution of rice flour affecting the starch enzymatic hydrolysis and hydration properties. Carbohydr. Polym..

[72] Fois S., Tolu V., Sanna V., Loddo A., Sanna M., Piu P.P., Piras D., Roggio T., Catzeddu P. (2025). Valorizing Carasau Bread Residue through Sourdough Fermentation: From Bread Waste to Bread Taste. Microorganisms.

[73] Peers F.G. (1953). The phytase of wheat. Biochem. J..

[74] Reale A., Di Stasio L., Di Renzo T., De Caro S., Ferranti P., Picariello G., Addeo F., Mamone G. (2021). Bacteria do it better! Proteomics suggests the molecular basis for improved digestibility of sourdough products. Food Chem..

[75] Di Stasio L., De Caro S., Marulo S., Di Renzo T., Ferranti P., Reale A., Mamone G. (2025). Impact of Microbial Leavening Agents and Fermentation Time on the In Vitro Digestibility of Neapolitan Pizza. Foods.

[76] Del Pino-García R., García-Lomillo J., Rivero-Pérez M.D., González-SanJosé M.L., Muñiz P. (2015). Adaptation and validation of QUick, easy, new, CHEap, and reproducible (QUENCHER) antioxidant capacity assays in model products obtained from residual wine pomace. J. Agric. Food Chem..

[77] Re R., Pellegrini N., Proteggente A., Pannala A., Yang M., Rice-Evans C. (1999). Antioxidant activity applying an improved ABTS radical cation decolorization assay. Free Radic. Biol. Med..

[78] Sanna M., Sanna S., Serra M., Roggio T., Catzeddu P., Sanna V. (2026). From Composition to Acceptance: Linking Nutritional, Structural and Sensory Attributes in Clean-Label Breads. Foods.

[79] Benzie I.F.F., Strain J.J. (1996). The Ferric Reducing Ability of Plasma (FRAP) as a Measure of “Antioxidant Power”: The FRAP Assay. Anal. Biochem..

